# Caffeinated Alcoholic Beverages – An Emerging Trend in Alcohol Abuse

**DOI:** 10.4172/2155-6105.S4-012

**Published:** 2013-08-20

**Authors:** Kelle M Franklin, Sheketha R Hauser, Richard L. Bell, Eric A Engleman

**Affiliations:** Institute of Psychiatric Research, Department of Psychiatry, Indiana University School of Medicine, Indianapolis, IN 46202, USA

**Keywords:** Alcohol, Caffeine, Caffeinated alcohol, Energy drink, Adenosine, Dopamine, Alcohol-preferring rat

## Abstract

Alcohol use disorders are pervasive in society and their impact affects quality of life, morbidity and mortality, as well as individual productivity. Alcohol has detrimental effects on an individual’s physiology and nervous system, and is associated with disorders of many organ and endocrine systems impacting an individual’s health, behavior, and ability to interact with others. Youth are particularly affected. Unfortunately, adolescent usage also increases the probability for a progression to dependence. Several areas of research indicate that the deleterious effects of alcohol abuse may be exacerbated by mixing caffeine with alcohol. Some behavioral evidence suggests that caffeine increases alcohol drinking and binge drinking episodes, which in turn can foster the development of alcohol dependence. As a relatively new public health concern, the epidemiological focus has been to establish a need for investigating the effects of caffeinated alcohol. While the trend of co-consuming these substances is growing, knowledge of the central mechanisms associated with caffeinated ethanol has been lacking. Research suggests that caffeine and ethanol can have additive or synergistic pharmacological actions and neuroadaptations, with the adenosine and dopamine systems in particular implicated. However, the limited literature on the central effects of caffeinated ethanol provides an impetus to increase our knowledge of the neuroadaptive effects of this combination and their impact on cognition and behavior. Research from our laboratories indicates that an established rodent animal model of alcoholism can be extended to investigate the acute and chronic effects of caffeinated ethanol.

## Prevalence and Impact of Alcohol use Disorders

Extensive evidence indicates that alcohol abuse has widespread social, economic, behavioral, and physiological consequences [1]. The Centers for Disease Control and Prevention (CDC) rank alcohol abuse as the third leading cause of preventable death [2]. For example, a causal relationship has been suggested between alcohol abuse and at least 50 different medical conditions [3]; also see [4] for a discussion on the role of genetics). According to the National Highway Traffic Safety Administration [5], nearly one-third of traffic fatalities in the United States involve at least one vehicle operator with a blood alcohol level (BAL) of at least 0.08 gram %, which is the legal BAL threshold for driving while impaired in the United States [6]. Direct and indirect costs of alcohol abuse have been estimated to approximate $500 billion annually [7]. Over half of adult Americans have a close relative with an alcohol use disorder (AUD), and a subset of these individuals have this trait across multiple generations.

The gap between men and women in the prevalence of AUDs and episodes of intoxication has been decreasing among youth and the elderly [8,9]. Regardless of gender, recent years have witnessed an increase in alcohol-related problems, particularly in young drinkers [10–12]. This is a serious concern, as early onset of alcohol use is a significant risk factor in the development and time-course of alcohol dependence [13,14].

Binge drinking similarly has been associated with future alcohol dependence problems and with younger drinkers [15–17]. More than 70% of surveyed college students report having engaged in alcohol binge drinking during their high school years [17]. Alcohol drinking in underage drinkers aged 12–20 years accounts for 11% of alcohol consumption in the United States, with a majority of this intake occurring during binge drinking episodes [18]. Regarding post-college-age binge-drinking, 45% of Americans aged 21–25 report that they had engaged in alcohol binge drinking in the previous month [19]. Nearly half of all individuals meeting life-time diagnostic criteria for alcohol dependence do so by the age of 21 and this increases to approximately two-thirds by the age of 25 [20]. In summary, the association of binge drinking with the development of alcohol dependence is magnified by the propensity of young people to participate in this behavior.

## Caffeine in the Context of Alcohol Abuse

Further complicating this issue is the common occurrence of consuming others drugs with alcohol. Polydrug use may introduce unique, complex cognitive and behavioral interactions that cannot be addressed by assessing the effects of each drug individually. Alcoholic populations exhibit an extremely high rate of poly-drug use [21]. One substance that is increasingly paired with alcohol is caffeine [22].

Caffeine (1,3-trimethylxanthine) is the United States’ most used psychoactive substance [23,24]. Caffeine largely has been eschewed as a psychoactive drug, despite, or perhaps because of, its widespread use. The U.S. Food and Drug Administration (FDA) have categorized caffeine as an ingredient “generally recognized as safe” [22]. However, it is a mild psycho stimulant, with motor effects similar to more potent drugs of abuse, such as cocaine and amphetamine [25,26]. Despite basic and clinical evidence that caffeine is reinforcing [tolerance can develop to some of its effects, and cravings and mild withdrawal symptoms can ensue after cessation of consumption; [25,27–30], it is rarely considered a drug of abuse. Nevertheless, if dependence criteria, in their broadest sense, from the fourth edition of the Diagnostic and Statistical Manual of Mental Disorders Text Revision (DSM-IV-TR) [31] are used, a significant portion of the population would be considered dependent upon caffeine intake [32,33]. What is especially troubling is that, similar to alcohol, adolescents and young adults are especially prone to abuse caffeinated beverages [34–36].

Behavioral and genetic associations indicate that there is a significant link between caffeine and alcohol intake [37–42]. Regarding caffeine abuse by alcoholics, Zeiner et al. [43] reported that alcohol-dependent individuals consume approximately 30% more caffeine daily, compared to their non-alcoholic counterparts. In addition, reports suggest that detoxified alcoholics consume twice as much coffee following cessation of alcohol drinking, compared to their intake prior to treatment [44]. This could be a serious concern for treatment-seeking alcoholics. For example, using caffeine intake as a substitute stimulus for alcohol consumption could interfere with psychological and physiological efforts to overcome addiction-related behaviors. Further, it is unclear what impact, if any, a history of heavy alcohol drinking could have on caffeine’s pharmacological profile, and whether this could affect the caffeine levels consumed by actively drinking and detoxified alcoholics. Assuming that caffeine and ethanol share at least some mechanisms of action, it is possible that physiological adaptations could generalize between the two substances. In addition, this elevated caffeine intake by alcoholics could be indicative of a more generalized physiological or genetic vulnerability to substance abuse and could mandate specialized treatment strategies.

In support of this latter suggestion, an assessment of adult twins indicates that ethanol and caffeine use disorders reflect approximately 50% heritability, although genetic loading for caffeine dependence may be lower than that for alcoholism [45–47]. In addition, poly-drug twin study analyses have reported a significant heritability effect on the co-use of ethanol and caffeine [48–50]. Similarly, Svikis et al. [51] reported that caffeine-dependent women who were family history positive (FHP) for alcoholism were less successful in reducing caffeine intake during pregnancy, relative to those who were family-history negative (FHN) for alcoholism. These authors [51] concluded that FHP individuals may require more intensive dependence intervention, due to a generalized vulnerability to heavy substance use. Within the context of dual-diagnosis, it is important to recognize that Bergin and Kendler [52] reported significant correlations (genetic and/or environmental) between caffeine use, tolerance and/or withdrawal and generalized anxiety, panic, phobic as well as major depressive disorders, conditions which also have been associated with heavy alcohol use.

Behavioral genetic reports indicating that a family history of alcoholism affects caffeine intake coincide with some pre-clinical findings in our laboratory. We performed experiments to investigate whether rats selectively bred for high alcohol preference also would display greater caffeine intake, relative to their progenitor stock. Alcohol-preferring (P) rats satisfy the criteria proposed for animal models of alcoholism, including consuming alcohol for its pharmacological effects and exhibiting signs of alcohol dependence and tolerance [53,54]. The genetically selected P rat shares some of the characteristics of FHP individuals, including early onset of excessive alcohol intake, lower sensitivity to the high-dose effects of alcohol and greater sensitivity to the behavioral and autonomic stimulating effects of low-dose alcohol, compared with NP (FHN) and outbred Wistar rats [55,56]. These rats exhibit neural and behavioral differences, as compared to nonpreferring NP counterparts and non-selected rats [57,58], such as performance of binge-like alcohol drinking behaviors [59–61]. P rats obtain pharmacologically relevant blood alcohol levels under several alcohol access conditions [57,59,61,62], and evidence supports the use of these animals to model human binge drinking [63].

Also similar to human alcoholics, P rats and other rodent models of alcoholism exhibit a greater general preference for some rewarding stimuli, such as illicit drugs, including nicotine [64] and 3,4-methylenedioxymethamphetamine (ecstasy; [65], sucrose, and novelty, relative to non-preferring and non-selected rodent lines [e.g., 66–68]. Previous studies have reported that subjects selectively bred for high alcohol intake exhibit greater behavioral and neurochemical responses to psycho stimulants, compared to low alcohol drinking or non-selected lines. Hyyatiä and Sinclair [69] reported that high alcohol-preferring Alko Alcohol (AA) rats consume more cocaine orally than do their alcohol non-preferring Alko, non-Alcohol (ANA) counterparts. Similarly, Wistar and AA rats behaviorally selected for high alcohol consumption exhibit increased conditioned place preference for cocaine [70,71], compared to their low drinking counterparts. Further, greater increases in cocaine-induced extracellular DA release in striatal compartments have been reported in Sardinian alcohol-preferring (sP) and AA rats, compared to alcohol non-preferring subjects [72,73]. Taken in conjunction with findings that high alcohol-preferring rat lines exhibit differential consumption of and neurobehavioral responses to other drugs of abuse beyond alcohol, such as cocaine and nicotine, compared to nonpreferring and nonselected counterparts, it is likely that these animals also could provide a useful model to examine caffeine intake.

Regarding the association of a genetic background for high alcohol preference with caffeine intake, we assessed female P rats and outbred Wistar rats for free-choice 24-hr intake of multiple concentrations (0.3 and 1.0 mg/ml) of caffeine and water over a 7-week period. These concentrations were selected from those used in a previous caffeine intake study [74]. From the human perspective, these concentrations amount to the difference between a 16-oz. serving of coffee from McDonald’s^®^ or Starbucks^®^, respectively [75]. We hypothesized that P rats would consume more total caffeine per day, vs. Wistar rats, and that the greatest differences would occur at the 1.0 mg/ml caffeine concentration.

**Figure 1:**
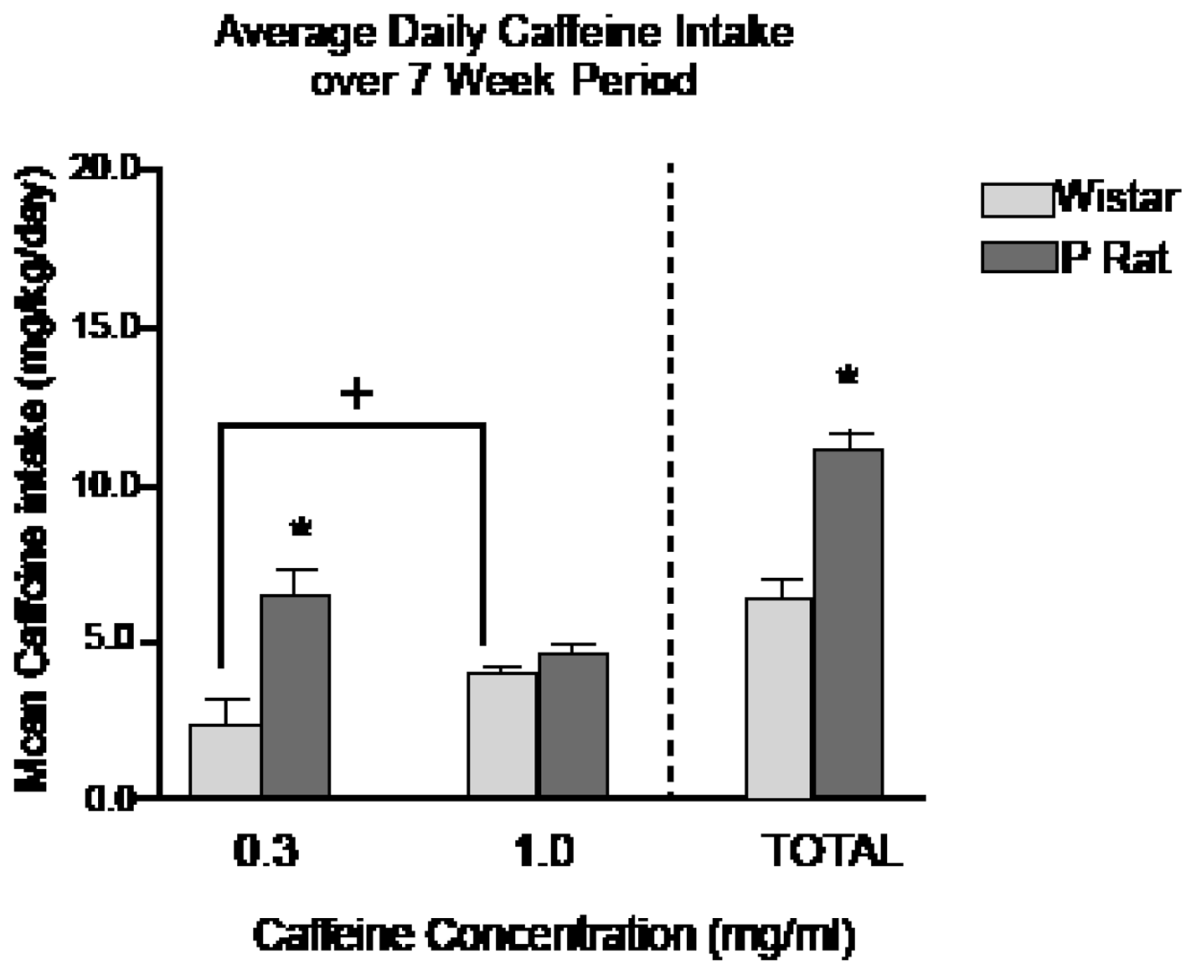
Daily caffeine intake for female Wistar (light gray bar) and P (dark gray bar) rats given 24-hour continuous access to multiple caffeine concentrations (0.3, and 1.0 mg/ml), with food and water available ad lib (n = 12/line).The total amount reflects the sum of daily intake of 0.3 and 1.0 mg/ml caffeine. ^*,^ indicates p < 0.05 vs. Wistar; ^+^, indicates p < 0.05 vs. 0.3 mg/ml caffeine concentration.

At no time in the course of this study did either P or Wistar groups exhibit a preference for caffeine solution over water. It has been shown previously that a period of forced access to caffeine is needed to observe caffeine preference over water [76]. The results indicated that P rats consumed more total caffeine (mg/kg) per 24-hr period than Wistar rats, such that consumption levels were approximately 10.5 vs. 6.5 mg/kg/24 hr, respectively (Figure 1). Clinically, reports indicate that non-dependent humans consume about 4–5 mg caffeine/70 g body weight. In contrast to our a priori hypothesis, the majority of this difference was associated with the lower (0.3 mg/ml) caffeine concentration, whereas intake of the higher (1.0 mg/ml) concentration was approximately equal in the two rat lines. One possible explanation for this finding is that intake of the higher concentration may have been limited by its flavor profile in both rat lines. In contrast, the two rat lines may have exhibited differential intake of the lower caffeine concentration due to the pharmacological properties of the compounds. Similar line differences for concentration-dependent differences in response to alcohol have been reported previously [77]. Overall, P rats consumed more caffeine than did outbred Wistar rats. This difference suggests that genetic selection for high alcohol preference also may have generated a propensity for elevated caffeine intake. These findings may provide additional validity for the use of the P animal model of alcoholism to examine ethanol co-abuse with other substances. Furthermore, this study provides basic research support for clinical findings indicating an association between alcohol and caffeine intake, which may be mediated by common reward neurocircuitry.

## Caffeinated Alcoholic Beverages

The introduction of Red Bull^®^ energy drink in the United States in 1997 sparked a surge in consumption of “energy drinks,” as well as energy drinks mixed with alcohol [78]. These products essentially combine purported performance-enhancing ingredients (e.g., guarana, taurine, ginseng, green tea extract, and/or B-complex vitamins) with high doses of caffeine (55 to 505 mg/serving) [78]. In comparison, 12- oz cans of Coca-Cola and Mountain Dew soft drinks contain 34 and 54 mg caffeine, respectively [79,80]. As a parallel to binge alcohol-drinking, approximately one-third of 12–24 year-olds consume energy drinks on a regular basis [81]. A reported decline in soft drink consumers between 2003 and 2008 likely reflects, in part, a doubling in the number of energy drink consumers during that same time frame [82].

As indicated above, an alarming trend is the addition of alcohol to these energy drinks. In general, marketing strategies for energy drinks have targeted young males [78], a group which also exhibits the greatest prevalence and frequency of alcohol binge drinking [83]. This is particularly of concern due to the lack of available information that addresses whether a history of heavy caffeine intake affects alcohol-related pharmacology and toxicology. With the wide acceptance of energy drinks, more and more drinking establishments and manufacturers have increased the caffeine content of their alcoholic beverages. The growing availability of high-caffeine energy drinks and pre-caffeinated alcoholic drinks has translated into high levels of caffeine and ethanol co-consumption. Young drinkers are the target market for many of these cocktails [84] and have contributed to their popularity [85]. Particularly concerning is that caffeinated alcoholic beverages may initiate earlier and more extreme caffeine and alcohol intake in younger populations. A 2006 web-based survey of ten colleges in North Carolina found that 24% of students who reported alcohol intake in the prior month had mixed alcohol with an energy drink [86]. Another sample of U.S. college students indicated that nearly half of survey respondents had consumed alcohol mixed with energy drink [84]. In Brazil, more than three-quarters of survey participants reported regular consumption of energy drinks mixed with alcohol [87], whereas in Turkey, the incidence in college students has been reported at 40% [88].

The popularity of these beverages has encouraged researchers to question what motivates their consumption. A standardized assessment of the impact of expectancies on motivation to consume these beverages indicated that caffeine was believed to enhance alcohol-related intoxication, a factor also associated with increased intake of the mixtures [89]. Caffeine and energy drinks have been reported to reduce ethanol-induced sedation or perception of sedation in humans and animals [87,90–92], although these findings have been mixed [93]. Consumption of caffeinated alcoholic beverages also increases reports of happiness and euphoria, behavioral disinhibition, and physical vigor, relative to alcohol alone [87,94].

However, just as individuals who consume large amounts of alcohol often underestimate how much alcohol affects them [95], increasing evidence indicates this may be compounded when these individuals consume caffeinated alcoholic beverages. Despite indications that young consumers of these drinks do not discount the risks of alcohol-related negative consequences when caffeine is co-consumed [89], some evidence in the U.S. and Canada indicates that adding caffeine to alcohol actually may increase these risks. Several reports indicate that caffeine co-administration increases alcohol intake and hazardous alcohol drinking [86,94,96,97]. Co-consumption of energy drinks and alcohol has been reported to triple the likelihood of binge alcohol drinking, relative to drinking alcohol alone [96]. O’Brien et al. [86] found that students who mixed alcohol and caffeine reported more heavy alcohol drinking episodes and twice as many episodes of weekly intoxication. A recent self-report study found that high-frequency energy drink consumers (>1/week) were heavier alcohol drinkers, drank alcohol more often, had greater risk for alcohol-related problems, and exhibited a higher risk of meeting DSM-IV criteria for alcohol dependence, relative to those with low or no energy drink consumption [97], but see comment by Skeen and Glenn [98]. Several reports indicate that consumers of caffeinated alcoholic beverages engage in more violent and risky behaviors, and experience more negative consequences, compared to those drinking alcohol alone. Particularly concerning are reports that consumers of caffeinated alcoholic beverages evidence more assaults (as perpetrators or victims), automobile incidents, and planned and actual alcohol-impaired driving episodes, relative to those drinking alcohol alone [86,99–101]. These negative consequences are particularly evident in adolescent populations [102].

It has been postulated that caffeine reduces perceived ethanol intoxication with little or no change to the cognitive impairing effects of ethanol [103,104], although this decrease may be task-dependent [105,106]. Expectations also could be involved in these findings. Evidence indicates that psychomotor tolerance to ethanol is increased in humans with a prior history of combining caffeine and ethanol, compared to individuals who have experienced either drug alone [107]. Moreover, this tolerance likely is, in part, an unconscious cognitive construct. Fillmore et al. [107] reported that informing subjects that caffeine would interfere with ethanol-induced sedation diminished caffeine’s ability to do so, and Fillmore [108] suggested that the expectation that caffeine would interfere with ethanol-induced sedation could trigger compensatory physiological mechanisms to maintain the disruptive influence of ethanol. These results emphasize the role of cognitive processes in the physiological response to ethanol in human subjects.

With regard to motor functioning, Marczinski and Fillmore [109] and Marczinski et al. [110] presented evidence that caffeine attenuates ethanol-induced motor skill disturbances. In cued go/no go task, consuming an energy drink along with ethanol was reported to improve reaction time for identifying and providing a keyboard response for a “go” visual target (i.e. a green rectangle), but failed to attenuate ethanol-induced reductions in inhibitory control, in response to a “no-go” target (i.e. a blue rectangle) [109,110].

Marczinski et al. [111] recently reported that the co-administration of the energy drink Red Bull^®^ with alcohol did not alter the ethanol-induced impairment on objective measures such as dual-task information and motor coordination but reduced perceptions of mental fatigue and enhanced feelings of stimulation compared to alcohol alone. These results may suggest that the combination of ethanol with caffeine could lead to inaccurate perceptions of performance abilities [111]. This finding is particularly disturbing in light of the increased propensity to engage in alcohol-impaired driving following caffeinated alcohol intake, relative to alcohol alone [96,101], as well as findings which indicate that caffeine does not attenuate alcohol-related impairment in simulated driving or sustained attention/reaction time [112]. Overall, caffeine likely exerts stimulatory effects to counter ethanol’s sedative properties, but does not alter ethanol-induced behavioral disinhibition, which could contribute to increased risk-taking during impaired driving. Similarly, Ferreira et al. [87] reported that subjective reports of physiological states and behavioral abilities following consumption of Red Bull^®^ energy drink and 37.5% v/v vodka co-administration did not correspond to objective behavioral measures of intoxication. Participants in this study reported reduced headache, weakness, dry mouth, and motor impairment following the co-administration of an energy drink and alcohol. However, measurements of motor coordination and visual reaction time indicated no differences between individuals consuming alcohol with or without the addition of the energy drink [87].

While some behavioral data have been used to conclude that caffeinated alcohol is pharmacologically differentiable from alcohol alone, existing behavioral support for this stance is co relational. These reports could be overlooking intervening variables that link caffeinated alcoholic beverage intake with high levels of alcohol consumption, such as high sensation-seeking personality [32] or age. It also is noteworthy that many studies that report detrimental effects of mixing alcohol and caffeine were reported in the U.S. or Canada, and could reflect social or cultural norms. As indicated above, a survey of young adults in Brazil and Turkey found 75% vs. 40% engaged in caffeinated alcohol beverage consumption, respectively [87,88]. Recent reports from The Netherlands and Australia suggest that energy drink consumption actually reduces alcohol drinking [113] and/or negative alcohol-related consequences [94,113]. Thus, it currently is unclear whether caffeine’s apparent exacerbation of heavy alcohol drinking reflects a physiological effect or a loading of social and demographic risk factors such as race, age, gender, culture, religion, and involvement in the college Greek community, which can influence the propensity to consume large amounts of caffeinated alcoholic beverages [86]. Regardless of whether caffeine-facilitated elevations in alcohol intake are social or biological phenomena, the long-term consequences of this behavior are cause for concern [22].

Besides the growing concern from the scientific research detailing risks associated with consumption of caffeinated alcoholic beverages reports of several deaths and hospitalizations following consumption of this drug combination have increased the focus on the potential dangers. In response to evidence that caffeinated alcohol intake may be harmful, the FDA proclaimed caffeine to be an “unsafe food additive” when combined with alcohol [22]. The primary explanation for this characterization was due to caffeine’s ability to mask some indicators of alcohol intoxication, without exhibiting clear effects on alcohol metabolism [22,114]. Warning letters from the FDA to 4 caffeinated alcoholic beverage manufacturers questioned the safety of caffeinated alcoholic beverages, and prompted several distributors to withdraw their products from the market [22]. However, it is unclear what impact this move will have on caffeinated alcohol intake in general, as reports suggest that the majority of these beverages are mixed by consumers, rather than being caffeinated prior to retail sale [89]. Further, an overall dearth in the body of research investigating the neurobehavioral effects of caffeinated alcoholic beverages weakened this warning and precluded a definitive statement regarding the relative safety of these mixtures.

**Figure 2:**
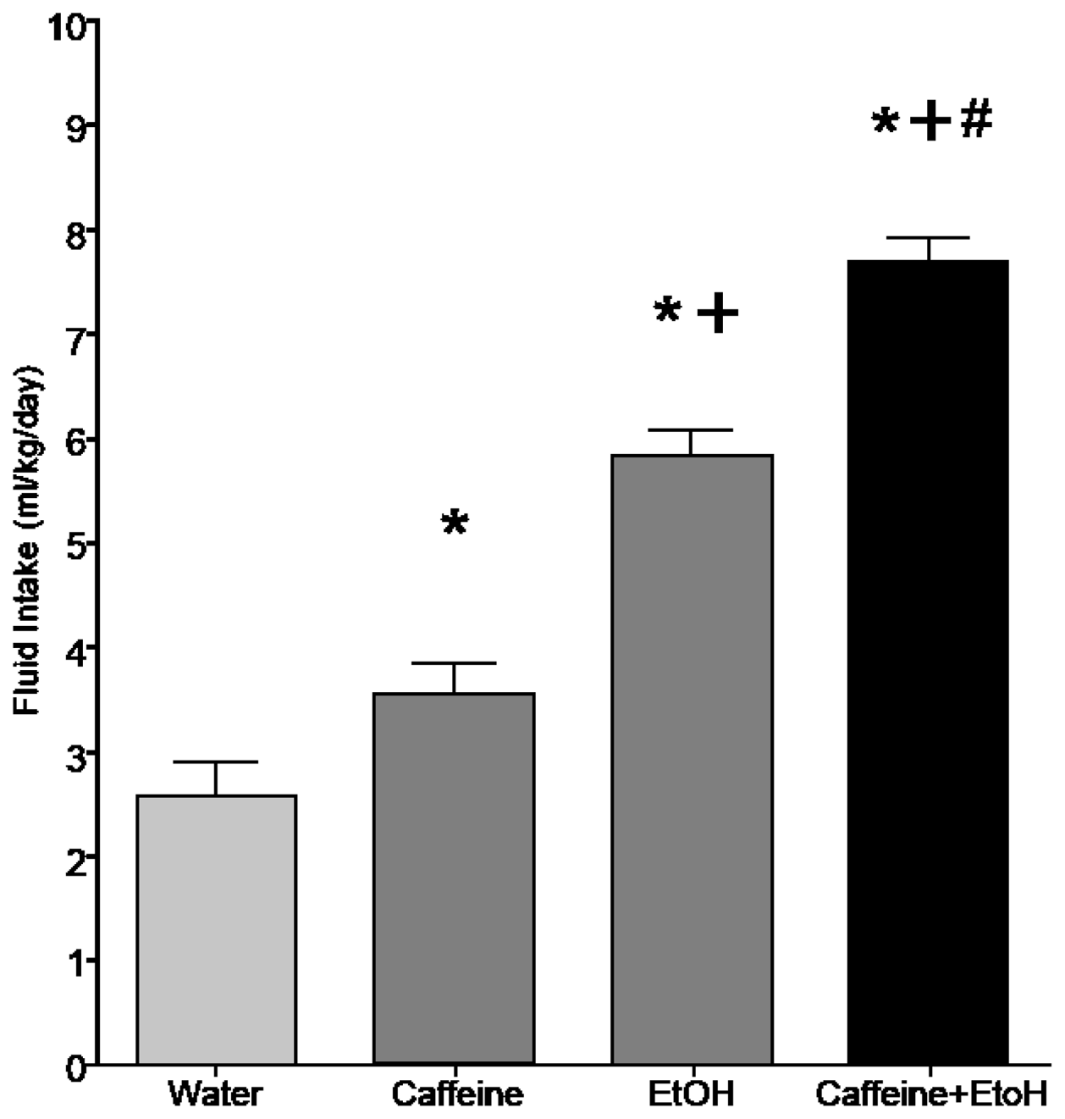
Average daily fluid intake (ml/kg) over 2 weeks of 1-hour/day limited access drinking sessions by adult female P rats (n = 18–30/group). ^*^ indicates p < 0.05 vs. water; ^+^ indicates p < 0.05 vs. 0.3 mg/ml caffeine; ^#^ indicates p < 0.05 vs. 15% v/v ethanol.

Findings that alcoholics and individuals with increased vulnerability for developing AUDs also consume more caffeine confirm a need to investigate whether alcohol and caffeine co-consumption increases alcohol intake and/or increases the likelihood of developing AUDs (e.g. [97]). Young people, in particular, have exhibited an attraction for consuming caffeinated alcoholic beverages, and often engage in binge drinking this combination. An extensive literature highlights the deleterious effects, both peripherally and centrally, of binge alcohol-drinking [86]. Given that the addition of caffeine to alcoholic beverages appears to exacerbate binge-drinking, it is absolutely necessary that research into the acute and long-term effects of this combination be undertaken immediately. Following our previous findings that P rats consume more caffeine than do Wistar rats (Figure 1), as well as existing research detailing differences in ethanol intake between the two rat lines [58], our laboratory sought to examine levels of caffeinated ethanol intake in a controlled basic research environment. To that end, female P rats were assessed for intake of a caffeinated alcoholic solution, or one of its component ingredients: water, 0.3 mg/ml caffeine, or 15% ethanol over a 14-day 1-hr/day scheduled access period. In line with our a priori hypotheses, the results indicated that caffeinated ethanol intake was significantly greater than intake of water, caffeine, or ethanol alone (Figure 2). In addition, only the rats consuming caffeinated ethanol evidenced progressive increases in alcohol intake over the 14- day access period, which may signal a transition from casual drinking behaviors to dependence (data not shown). These findings may exhibit sub-additive or synergistic interactions of the two compounds, which may alter their rewarding or behavioral properties. However, without further investigation, it is unclear what neurobehavioral alterations are reflected by the increased intake of the combined compounds. In addition, these experiments were performed in female rats; however, sex-specific differences in self-administration are an important consideration. Future directions should look toward repeating these experiments in males to ascertain whether they would consume similar amounts of the solutions.

Overall, the findings indicate that caffeine elevates ethanol intake and, conversely, ethanol increases caffeine intake, even in the absence of human cultural biases. Generalized increases in activity due to the activating effects of caffeine and/or ethanol [115–119] could contribute to the increase in alcohol intake when caffeine is co-consumed. Elevations in drinking as a result of locomotor stimulation may support the common assumption that caffeine attenuates or masks the sedating effects of alcohol [91], which may enable individuals to consume more caffeinated alcohol drinks over a longer period of time [120].

Some animal research supports findings that caffeine attenuates the behavioral sedation associated with ethanol. El Yacoubi et al. [121] reported that 25 mg/kg caffeine decreased the duration of ethanol-induced loss of righting reflex in mice. Ferreira and colleagues [92] presented evidence that caffeinated energy drinks attenuate the locomotor-depressing effects of ethanol, such that 10.71 ml/kg Red Bull^®^ significantly decreased the sedating effects of 2.5 g/kg ethanol in Swiss mice. It is possible that combined mechanisms of caffeine and alcohol are involved in these findings. For example, caffeine can inhibit benzodiazepine binding to GABA_A_ receptors [122]. This is especially noteworthy/concerning because benzodiazepines are commonly used during alcohol detoxification [123], despite their high abuse/co-abuse potential [124]. These effects result primarily from caffeine blockade of adenosine receptors on striatal GABA neurons [119]. Blockade of VTA GABA_A_ receptor activation previously was posited to increase ethanol intake by reversing the drug’s GABA-mediated sedative-hypnotic effects [125].

In line with this research, Sudakov et al. [126] reported that chronic caffeine drinking increases sensitivity to the locomotor stimulating properties of ethanol, particularly in ethanol-insensitive subjects. As motor stimulation previously has been associated with low-dose ethanol preference and reinforcement [118], this suggestion may indicate that caffeine facilitates ethanol drinking by increasing sensitivity to reward-inducing psychomotor effects associated with ethanol intake.

## Pharmacokinetic Interactions of Alcohol and Caffeine

The possibility that caffeine increases alcohol intake due to changes in ethanol’s pharmacokinetic properties has prompted several laboratories, including our own, to pursue this line of research. Caffeine increases metabolic rate, both in active and sedentary individuals [127]. It is plausible that the thermogenic or diuretic properties of caffeine [127–129] could facilitate ethanol elimination through increases in transdermal evaporation or water and sodium excretion. However, according to Ferreira et al. [87,92] and Kunin et al. [130], caffeine exposure does not alter ethanol pharmacokinetics in humans or rodents to a significant degree.

Ferreira et al. [87] reported that the addition of Red Bull^®^ energy drink did not alter breath alcohol concentration levels or alcohol elimination rates associated with 0.6 or 1.0 g/kg 37.5% v/v vodka, over a 150-min time course. Similarly, intragastric administration of a Red Bull^®^/vodka mixture did not reduce 30-min BALs in mice [92]. Kunin et al. [130] reported similar findings using male Wistar rats. These researchers [130] reported that caffeine pretreatment (i.p.), delivered 30 min prior to systemic ethanol injections (i.p.) did not alter BALs at 15 or 30 min following alcohol exposure [130].

Our laboratory has observed similar outcomes to those presented by Kunin et al. [130] and Ferreira et al. [87,92] with regard to peak ethanol levels. We utilized subcutaneous microdialysis [131] to assess the ability of caffeine to alter alcohol pharmacokinetics in female P rats. The time-course of interstitial ethanol levels was assessed, beginning immediately after acute exposure to 1 mg/kg caffeine dissolved in 0.5 g/kg 15% w/v ethanol, or 30 mg/kg caffeine dissolved in 2.0 g/kg 15% w/v ethanol, or equivalent doses of 15% w/v ethanol alone. In line with previous evidence, we hypothesized that co-administration of caffeine would not alter the BALs of P rats. In support of this hypothesis, peak ethanol levels were not altered by caffeine co-administration. However, we did observe some evidence that caffeine increases ethanol clearance at low doses (1 mg/kg caffeine; 0.5 g/kg ethanol; Table 1). Due to methodological differences, it is impossible to make direct comparisons with the results presented by Ferreira et al. [87], who did not observe caffeine-induced differences in ethanol clearance. Furthermore, this effect was not present with the higher dose combination, indicating that this alteration was low-dose specific. The apparent lack of effects at higher alcohol and caffeine doses does not support extrapolation of these data to explain some of the behavioral differences that have been reported following heavy consumption of caffeinated alcoholic beverages. In contrast, these data likely support previous reports that exclude differences in ethanol-related pharmacokinetics as a potential explanation for caffeine’s ability to alter the effects of ethanol.

With the exception of assessing alcohol levels, few studies deliver a concentrated effort to identify the neural and physiological mechanisms that might underlie purported behavioral and cognitive differences between alcohol drinking and caffeinated alcohol drinking. To that end, research that examines neural effects of caffeine or ethanol individually often is the only available tool to extrapolate the effects of these substances when consumed in combination.

## Overlapping Systems Associated with Caffeine and Ethanol: Adenosine and Dopamine

**Figure 3:**
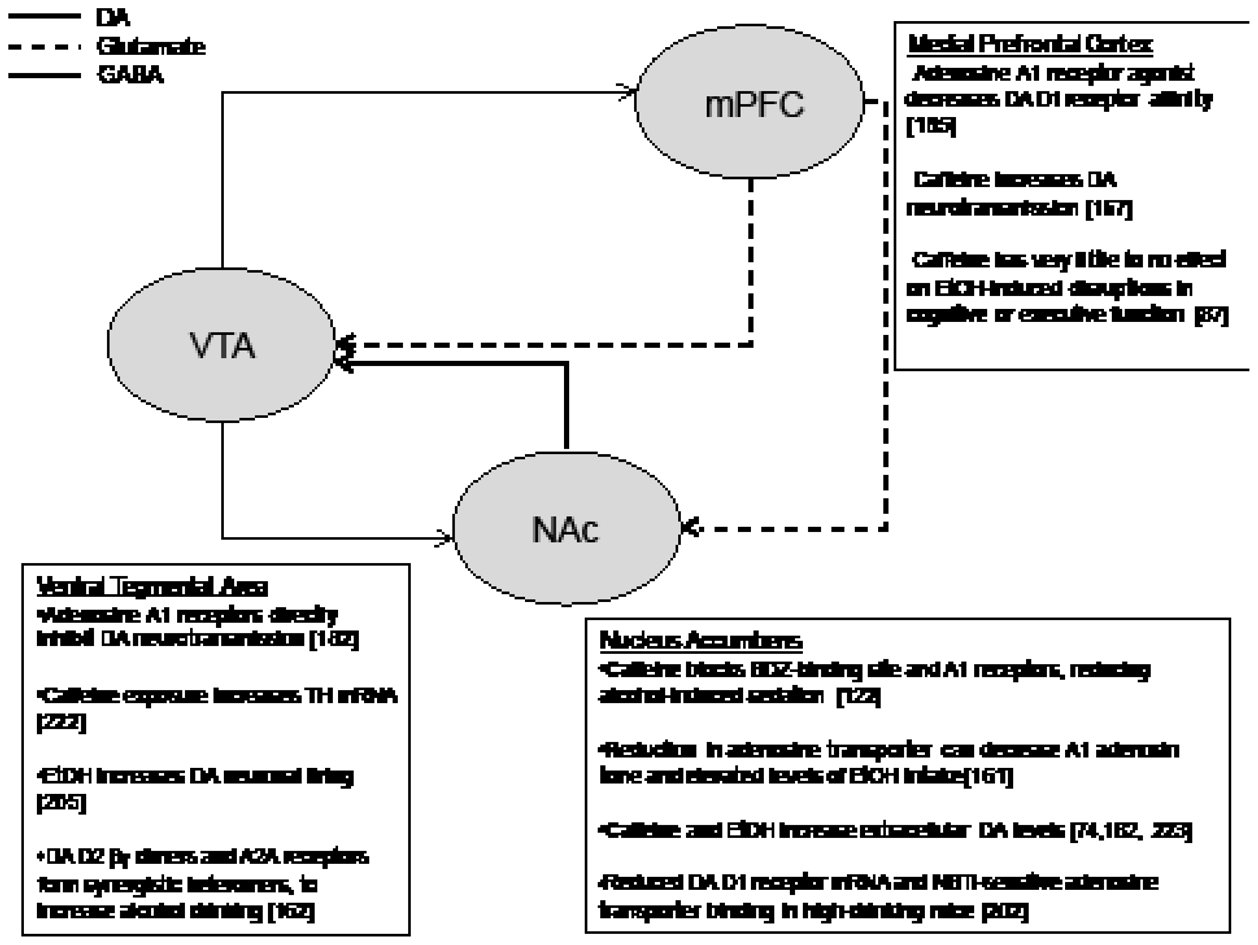
A representation of possible neurochemical/molecular sites of interaction between ethanol and caffeine. The MCL DA system includes afferent projections from DAneurons in the VTA to several projections regions, including the mPFC and NAc. Neurons of the mPFC are primarily glutamatergic, whereas the majority of neurons in the NAc are GABAergic medium spiny neurons. While evidence for the effects of caffeinated ethanol in this system is limited, it may be hypothesized that caffeine enhances EtOH’s effects, increasing DA and glutamate neurotransmission, while disinhibiting release of these transmitters through reductions in NAc GABA signaling. Taken together, co-administration of caffeine and ethanol may increase the rewarding and reinforcing properties associated with either drug alone. Repeated experience with this drug combination may initiate neural and/or behavioral adaptations. This may have important implications for the transition from recreational alcohol drinking to alcoholism, particularly in populations that exhibit greater vulnerability or predisposition to develop alcohol use disorders.

Due to the apparent behavioral and genetic interactions of AUDs and caffeine intake, an important area of research is to identify neural systems that underlie some of these interactions. Both caffeine and ethanol affect dopaminergic and adenosinergic neurotransmission, which are thought to contribute to their neurobiological and reinforcing properties. Figure 3 shows a schematic of dopamine, glutamate, and GABA neurotransmission in the MCL system, as well as providing selected evidence for the manner in which caffeine and ethanol exposure could affect these signals.

### Adenosine

Adenosine is a purine neurotransmitter/neuromodulator with many (largely inhibitory) central and peripheral sites of action mediated through four identified G-protein coupled receptors, A_1_, A_2A_, A_2B_, and A_3_ [132,133]. Adenosine receptors exhibit widespread central distribution and modulate the majority of neurotransmitter systems, either directly or through indirect modulation of amino acid neurotransmitters [134,135]. Adenosine A_1_- and A_3_-type receptors are cyclic adenosine 5′ monophosphate (cAMP)-inhibiting, while A_2A_- and A_2B_-type receptors are cAMP-stimulating [136]. The majority of research involving adenosine receptors has targeted the A_1_ and A_2A_ receptors, which are sufficiently sensitive for activation through tonic adenosine signaling.

A_1_ receptors are the most densely populated adenosine receptor subtype within the central nervous system, and are present in the hippocampus, cerebral and cerebellar cortices, hypothalamus, and some areas of the thalamus [137,138]. A_1_ receptors have the highest affinity for adenosine binding (70 nM concentration) [139]. They have been associated with physiological-behavioral functions, such as sleep, arousal, and anxiety [139]. These receptors modulate the actions of nearly all other neurotransmitters, including dopamine.

Adenosine A_2_ receptors have been subdivided into A_2A_ and A_2B_ receptor types. A_2A_ receptors have an adenosine binding affinity of 150 nM. These receptors are less widespread in the CNS, relative to A_1_ or A_2B_ receptors. A_2A_ receptors primarily are localized in the striatum, olfactory tubercle, nucleus accumbens (NAc), and other areas receiving significant dopamine innervation [139–141]. A_2_ receptors are linked to adenylyl cyclase stimulation; their primary function is to regulate neurotransmitter release [142]. For further reviews on adenosine, see [143,144].

#### Adenosine and caffeine

Caffeine is a relatively nonspecific competitive antagonist of adenosine receptors. Adenosine Reports indicate that chronic caffeine exposure may reduce the compound’s ability to block A_1_ receptors, due to conformational changes in the receptors or endogenous ligand activity [74,145,146].

There has been a great deal of inconsistency in reports associated with the effects of caffeine intake on adenosine A_2A_ receptors. Some reports indicate that caffeine exposure does not have a lasting effect on adenosine A_2A_ receptors or their pharmacological modulators [147–149]. However, others have indicated that chronic caffeine experience up-regulates [150,151] or down-regulates [152] A_2A_ receptors. These results largely depend on the tested species and the employed caffeine exposure procedures.

#### Adenosine and ethanol

While the availability of research that examines caffeinated alcohol is limited, caffeine’s primary pharmacological mechanism of action occurs through direct antagonism of adenosine receptors. Therefore, evidence for direct interactions of adenosine and ethanol potentially could provide useful information regarding interactions of caffeine and ethanol.

Ethanol drinking has been reported to increase A_1_ receptor number [153], although chronic exposure also might desensitize these receptors in a manner similar to caffeine [74,145]. Adenosine neurotransmission has been associated with some effects of acute and prolonged ethanol exposure, as well as ethanol withdrawal [154,155]. Chronic ethanol also increases extracellular CNS adenosine levels with brain region specificity [156]. Adenosine A_1_ receptors have been implicated in mediating the anxiolytic and motor impairing effects of ethanol [157,158], as well as withdrawal-induced seizures [154].

Ethanol’s effects also are associated with adenosine A_2A_ receptor signaling. Adenosine A_2A_ receptors contribute to ethanol-induced motor effects. For example, El Yacoubi et al. [121] found that A_2A_ knockout mice exhibit a shorter latency to regain righting reflex following acute ethanol exposure, relative to wild-type mice. Some pre-clinical research indicates that ethanol-induced reinforcement is reduced in subjects lacking or having diminished A_2A_ receptor functioning [159,160]. It has been reported that systemic administration of 3 and 10 [159] or 10 and 20 [160] mg/kg doses of the partially selective A_2A_ receptor antagonist 3,7-dimethyl-1-propargylxanthine (DMPX) dose-dependently reduced ethanol-reinforced operant responding. In contrast, a lower (1 mg/kg) dose of DMPX has been shown to increase ethanol-reinforced operant responding [160].

Evidence indicates that sedative and ataxic responses to ethanol also are reduced in mice lacking the equilibrative nucleoside transporter 1 [161]. This transporter is responsible for facilitating adenosine diffusion across cellular membranes. Acute ethanol exposure blocks these transporters [162–164], increasing extracellular adenosine levels, facilitating extracellular adenosine receptor activation, and inducing some of ethanol’s sedative-hypnotic effects. In contrast, chronic ethanol exposure likely desensitizes these transporters to ethanol blockade and contributes to desensitization of adenosine receptors [162–164]. The widespread central effects of ethanol and caffeine are not localized to adenosine systems only. They are manifested in multiple other neural circuits, including the mesocorticolimbic (MCL) dopamine system.

### Mesocorticolimbic dopamine system

The MCL dopamine system is comprised of dopamine cell bodies in the ventral tegmental area (VTA), and their projections to several forebrain areas, including the basolateral amygdala, hippocampus, lateral septum, olfactory tubercle, NAc, and the medial prefrontal cortex (mPFC). For a review on MCL dopamine activity, see [165].

Exposure to either caffeine or ethanol increases extracellular dopamine levels in MCL terminal regions [74,166–169], and stimulates feedback to the VTA to terminate dopamine output from this region [170–172]. Together, these findings suggest that caffeine and ethanol activate both inhibitory and excitatory neurocircuitry within the MCL system which may have implications for drug reward and abuse. From these and other reports, researchers have proposed that the role of adenosine receptors to modulate ethanol or caffeine reward likely is related, in part, to interactions with central dopaminergic systems.

#### Dopamine and adenosine in the mesocorticolimbic reward circuit

Adenosine signaling directly affects dopamine systems. Adenosine and dopamine interactions likely play integral roles in caffeine reinforcement and psychomotor stimulation. Adenosine A_1_ and A_2A_ receptors are found in many dopamine-rich areas of the central nervous system, including the MCL dopamine system [162,167,168]. Research indicates that adenosine and dopamine receptors form functionally interactive, primarily antagonistic heteromeric receptor complexes [173–179]. Similarly, caffeine-induced adenosine receptor antagonism alters dopamine neurotransmission. Acute high (10 mg/kg+) caffeine doses increase extracellular NAc dopamine levels [180], while lower doses [181] or prolonged exposure [74] have no such effect (but see [167]).

Quarta et al. [74] reported that prolonged (14 day) exposure to caffeine in the drinking water results in tolerance to the ability of caffeine or an A_1_ antagonist to increase extracellular NAc dopamine and glutamate levels [74]. In contrast, Borycz et al. [182] did not find that acute treatment with an A_1_ receptor antagonist altered extracellular mPFC dopamine levels. In line with reports from Quarta et al. [74], chronic modulation of adenosine receptors may be necessary to observe these neuroadaptations in the A_1_ receptor.

A_1_ receptors likely block some of the behavioral and neurochemical actions of D_1_ receptors. For example, pharmacological modulation of NAc and mPFC A_1_ receptors alters motor responses to D_1_ agonists and antagonists [183,184], as well as some D_1_-dependent behaviors [175,183]. In line with these findings, activation of A_1_ receptors desensitizes D_1_ receptors in striatal and limbic regions [178,185]. Similarly, adenosine A_1_ receptor blockade increases dopamine neurotransmission [145,177,186,187]. Evidence from cell lines and striatal regions indicates that A_1_ receptors drive the uncoupling of dopamine D_1_ receptors from stimulatory G-proteins and alter the conformation and binding affinity of MCL dopamine D_1_ receptors [175,177,185,186], which could account for some of the antagonistic influence that A_1_ receptors exert upon D_1_ receptors. Quarta et al. [145] reported that local perfusion with A_1_ receptor antagonist CPT (300 uM or 1 mM) increases extracellular NAc dopamine levels, although the range for CPT-induced alterations of dopamine neurotransmission may be concentration- and brain region-specific [182,188]. Overall, these findings suggest that A_1_ receptors play an inhibitory neuromodulatory role to diminish functional kinetics associated with dopamine D_1_ receptors. These effects likely represent some of the mechanisms through which adenosine receptor agonists induce sedation, and antagonists, such as caffeine, stimulate behavior [175].

Striatal _A2_ receptors largely are co-expressed with postsynaptic dopamine D_2_ receptors [189–191]. Research suggests that low density A_2_ receptor populations are sufficient to diminish the binding affinity of dense D_2_ receptor populations [192]. Constitutive populations of A_2_-D_2_heteromeric complexes have been identified [173,193]. Conformational changes resulting from A_2_ receptor activation are transmitted to D_2_ receptors [194], and may present a mechanism through which A_2_ receptor activation diminishes inhibitory D_2_ receptor signaling [179]. Moreover, A_2A_ receptor antagonists decrease dopamine tissue levels [195]. Previous studies have reported that a single injection of an A_2_ antagonist can decrease extracellular dopamine levels both in vivo and in vitro [145,196]. Similarly, Dassesse et al. [197] reported that mutant mice with diminished striatal A_2_ activity exhibit a concomitant decrease in extracellular dopamine levels, while mice lacking striatal D_2_ receptors exhibit impairments in A_2_ receptor-mediated functions [197]. In addition, A_2A_ receptor antagonists have been reported to counteract the cataleptic and tremor effects associated with D_2_ receptor blockade [195,198]. These effects may be mediated through A_2A_ receptor antagonist-induced conformational changes in D_2_ receptors and associated reductions in the binding affinity of D_2_- like receptor antagonists. Ultimately, these pharmacological alterations may result in desensitization of extracellular dopamine binding to excitatory neurons and decrease dopamine-modulated behavioral pathologies [195,198]. Taken together, these findings suggest that the A_2_ and D_2_ receptors interact to regulate adenosine and dopamine neurotransmission, as well as their resulting behavioral outputs.

A few initiatives have examined A_1_-D_2_ and A_2A_-D_1_ receptor interactions. Karcz-Kubicha [199] reported that co-activation of A_1_ receptors is necessary for A_2A_-mediated increases in c-fosimmunoreactivity in the mPFC anterior cingulate region [199]. These researchers [199] suggest that this permissive role for A_1_ receptors is related to their ability to block tonic dopamine neurotransmission and D_2_ receptor activation. A_2A_ and D_1_ receptor subtypes are not largely co-localized [200]; however, there apparently are some functional and behavioral interactions of the two receptor subtypes. For example, blockade of A_2_ receptors has been reported to potentiate the excitatory neurotransmission associated with dopamine D_1_ receptors [201]. In addition, A_2A_ and D_1_ receptor interactions have been reported to promote alcohol consumption [202]. However, overall, evidence indicates that A_2_ antagonists have greater ability to block D_2_ receptor-mediated effects, relative to those modulated by D_1_ receptors [193].

#### Dopamine and ethanol in the mesocorticolimbic reward circuit

There is substantial evidence for the involvement of the MCL dopamine system in ethanol drinking and reward. For example, neuroimaging experiments indicate that alcohol increases striatal dopamine neurotransmission in young adult males [203,204]. Similar pre-clinical findings have been reported in laboratory rodents. Pharmacologically relevant levels of ethanol have been reported to increase firing of VTA dopamine neurons [205,206]. In line with this, the VTA, NAc, and mPFC have been implicated in operant oral ethanol self-administration [207,208]. Rats will self-infuse ethanol directly into the VTA [209]. Local, peripheral and oral ethanol exposure potentiates extracellular dopamine levels in the NAc, likely leading to neuroadaptations in dopamine D_2_autoreceptor regulation of the circuit [169,210–214]. Similarly, naïve rats bred for high alcohol preference exhibit lower NAc tissue dopamine levels, compared to their alcohol non-preferring counterparts [169,215–217]. These findings suggest that differential dopamine neurotransmission in the MCL circuit could alter the reinforcing responses to ethanol exposure, and may represent a predisposing factor toward high ethanol intake and, by extension, possibly could increase the intake of caffeine, as well.

#### Dopamine and ethanol and adenosine in the mesocorticolimbic reward circuit

Adenosine modulation of MCL dopamine neurotransmission likely is involved in ethanol consumption. Despite the relatively low co-localization of A_2A_ and D_1_ receptors [200], evidence suggests that communication between these networks may be involved in alcohol drinking. Short et al. [202] selectively deleted A_2A_ and D_1_ receptors from mice to examine the involvement of these receptors in ethanol intake. Subjects with dual A_2A_ and D_1_ receptor knock-out significantly reduced their ethanol intake, relative to those with single-deletion or no alteration in the receptors [218]. In contrast, Naassila et al. [219] reported that A_2A_ receptor knock-out alone increases ethanol intake (but see, [220]). Differences in these studies may suggest that dopamine D_1_ receptors compensate for absent A_2A_ receptors and may implicate both of these receptor types in neural processing associated with alcohol drinking.

Yao et al. [162] found that A_2_ and D_2_ receptors synergize in the presence of ethanol to increase gene transcription via potentiation of PKA_Cα_ translocation to the cell nucleus. These researchers [162] reported that ethanol- and dopamine-induced increases in PKA_Cα_ translocation could be blocked by adenosine A_1_ and A_2_ receptor antagonists, in a manner mediated primarily by A_2_ receptors [162]. This synergy likely sensitizes the system to subsequent ethanol exposure, as well as endogenous dopamine D_2_ receptor signaling [162] and adenosine-mediated desensitization of Gα_s_-coupled receptors [164]. With prolonged elevation of extracellular adenosine levels (including that associated with chronic ethanol drinking [162]), A_2_ and D_2_ receptors uncouple, such that the inhibitory effects of D_2_ receptors predominate [221]. Chronic ethanol exposure has been reported to desensitize A_2A_ receptor response to agonists, as well as reduce the dopamine response to A_2A_ antagonism [162]. These reports present one mechanism through which A_2_ receptors may alter gene expression in a D_2_ receptor dependent manner. These effects may be potentiated by ethanol exposure. In line with this suggestion, Short et al. [202] reported differences in the NAc dopamine and adenosine systems of ethanol-naïve high alcohol-drinking C57Bl/6J (B6) mice and low alcohol-drinking CD-1 mice, which correlated with subsequent measures of ethanol intake. They reported decreases in D_1_ receptor mRNA and increases in D_2_ receptor binding in B6 vs. CD-1 mice. Short et al. [202] also reported that nucleoside transporter blocker nitrobenzylthioinosine (NBTI) binding was reduced in B6 mice, compared to the CD-1 line. This dysfunction would be expected to increase extracellular adenosine concentrations [162] and, under chronic conditions, may cause neuroadaptations, affecting adenosine receptor activity. These findings in rodent models suggest that a predisposition for high alcohol consumption may be correlated with decreases in intracellular adenosine transport.

## Conclusions

The negative impact of heavy alcohol use continues to be a public health concern. Binge alcohol drinking and early onset of alcohol use, in particular, appear to be risk factors for future alcohol dependence. Existing evidence suggests that the increased availability of caffeinated alcoholic beverages exacerbates both of these predispositions for developing alcohol dependence. This underscores the need for research examining the interactions of these two substances, on cognitive, behavioral, and neuronal/physiological function. The impact of caffeinated alcohol consumption has not been characterized fully; clinical evidence is mixed regarding whether caffeine increases alcohol intake and negative alcohol-related consequences in young drinkers, relative to alcohol alone. As with most substances of abuse, the interplay between abuse and factors such as gender, age, culture, social strata and religion is complex. However, pre-clinical evidence supports the contention that caffeine increases alcohol consumption, and animal models may provide new insights into central mechanisms that underlie the rewarding and reinforcing properties of this combination. The experimental data that we have presented from our laboratory were collected in a single genetic model of heavy and binge [60] alcohol intake, the P rat, during adulthood. However, future experiments should seek to evaluate systematically whether neural and behavioral evidence associated with intake of caffeinated alcohol solutions generalizes across different genetic populations. Findings from studies using different rodent models of alcoholism (e.g. B6 mice, P rats, AA rats, sP rats, etc.) and genetic mutant animal models can be expected to reveal important information on the central effects of this often abused drug combination. Given the increasing consumption of caffeinated alcoholic beverages in high-risk populations (e.g. adolescent binge-drinkers), future research should occur at multiple developmental time-points. Knowledge of the neurocircuitry and adaptations that are associated with acute and chronic caffeinated alcohol intake, as well as their long-range alterations following such consumption, will provide useful information regarding the intervention and treatment strategies for those who abuse this combination.

## Figures and Tables

**Table 1:** Peak ethanol levels (mg%) and ethanol elimination rate (mg%/min) in female P rats (n = 5–7/group) following acute i.p. exposure to 1 mg/kg caffeine dissolved in 0.5 g/kg 15% w/v ethanol (low), or 30 mg/kg caffeine dissolved in 2.0 g/kg 15% w/v ethanol (high), or equivalent doses of 15% w/v ethanol alone.

Treatment Condition	Ethanol Peak Level (mg%)	Ethanol Elimination(mg%/min)
Ethanol Low	48 ± 5	−0.70 ± 0.05
Ethanol/Caffeine Low	68 ± 6	−0.92 ± 0.14
Ethanol High	235 ± 10	−0.78 ± 0.17
Ethanol/Caffeine High	222 ± 19	−0.68 ± 0.02

* , indicates p < 0.05 vs. ethanol alone group.

## Abbreviations

MCL: Mesocorticolimbic
DA: Dopamine
VTA: Ventral Tegmental Area
mPFC: Medial Prefrontal Cortex
NAc: Nucleus Accumbens
GABA: γ-aminobutyric Acid
EtOH: Ethanol
TH: Tyrosine Hydroxylase
BDZ: Benzodiazepine
NBTI: Nitrobenzylthioinosine
